# Development and external validation of interpretable machine learning models for 7-, 14-, and 28-day mortality in sepsis-associated liver injury

**DOI:** 10.3389/fmed.2026.1942203

**Published:** 2026-10-16

**Authors:** Hao Yao, Zhaopu Li, Jinxiao Lü, Wei Shen

**Affiliations:** 1 Critical Care Medicine Department, Xiaogan Hospital Affiliated to Wuhan University of Science and Technology, Xiaogan, Hubei, China; 2 Medical College, Wuhan University of Science and Technology, Wuhan, Hubei, China

**Keywords:** external validation, machine learning, MELD-Na, mortality prediction, sepsis-associated liver injury, SHAP

## Abstract

**Background:**

Mortality in sepsis-associated liver injury (SALI) remains high, and early risk stratification is difficult. Existing prognostic models commonly focus on a single endpoint, usually 28-day mortality, and rarely undergo independent external validation.

**Methods:**

We developed machine-learning models using clinical features recorded during the first 24 h to predict all-cause in-hospital mortality within 7, 14, and 28 days. In MIMIC-IV (v3.1), 1,550 adults meeting the prespecified SALI criteria were allocated to a training cohort (n = 1,119) and a held-out internal validation cohort (*n* = 431). An independent Chinese cohort from Xiaogan Central Hospital (*n* = 229; 28-day in-hospital mortality, 45.9%) was used for external validation. One hundred candidate pipelines were evaluated using stratified 10-fold cross-validation. SHAP was used to describe feature attribution across prediction horizons.

**Results:**

AUROCs in the held-out internal validation cohort were 0.840, 0.841, and 0.850 for 7-, 14-, and 28-day mortality, respectively; corresponding external AUROCs were 0.720, 0.717, and 0.763. The external 28-day model outperformed modified SOFA (0.763 vs. 0.673; *P* = 0.011) and MELD-Na (0.763 vs. 0.636; *P* = 0.002). A parsimonious logistic-regression sensitivity model achieved an external 28-day AUROC of 0.762, not significantly different from the full model (*P* = 0.970). The 14-day model was miscalibrated externally; recalibration improved the calibration slope from 0.563 to 0.970 but did not materially improve the Brier score. SHAP rankings varied across horizons and were interpreted descriptively rather than causally.

**Conclusion:**

The models showed moderate transportability to one independent retrospective cohort. Multicentre prospective validation is needed before clinical implementation.

## 1 Introduction

Liver injury is one of the most common and lethal complications of sepsis. It occurs in 34%–46% of septic patients in some series, approximately doubles the risk of death, and is associated with 50%–68% of short-term mortality (1–5). The underlying biology is multifactorial, combining systemic inflammation, impaired hepatic microcirculation, oxidative mitochondrial injury, and direct hepatocellular damage (6, 7). Clinically, the central need is to identify which patients are most likely to die, and when, so that monitoring intensity and therapeutic effort can be matched to risk.

The SOFA score is widely used in intensive care (8) but underperforms in liver-specific subsets, and several studies have identified substantial room for improvement in sepsis prognosis through conventional scoring systems (9–11). Single biomarkers and standard logistic regression capture only part of the variance in SALI mortality. Machine learning approaches can model nonlinear interactions among clinical variables and therefore offer a plausible route to improved prediction (12–16). Two limitations nonetheless recur in the existing SALI literature. First, most studies target 28-day mortality as the sole endpoint and implicitly treat predictors as time-invariant, even when multiple timepoints are analysed (17). Second, most models are trained and validated within the same dataset (18), and external validation, particularly in non-Western populations, remains rare.

We addressed these gaps with a unified machine learning framework that predicts all-cause in-hospital mortality at three horizons (7, 14, and 28 days). Stratified 10-fold cross-validation was used to evaluate the candidate pipelines within the training cohort, followed by evaluation on a separate hold-out internal validation set and an independent external validation cohort. SHAP analysis was used to characterise model attribution within each prediction horizon. This study had three objectives: (i) to build and internally validate SALI mortality models for 7, 14, and 28 days from MIMIC-IV; (ii) to assess transportability in an independent Chinese cohort; and (iii) to describe, without causal attribution, how model-derived feature contributions vary across prediction horizons.

## 2 Materials and methods

### 2.1 Study design and data sources

This dual-cohort retrospective study is reported in accordance with the TRIPOD + AI statement (19); CONSORT-AI applies to reports of randomised trials of artificial intelligence interventions (20). The development cohort was derived from MIMIC-IV (v3.1) (21), which contains source data from 2008 through 2022. Sepsis was defined according to the Sepsis-3 framework as implemented in the source cohort extraction workflow (22): Suspected infection was identified by the combination of blood culture orders and intravenous antibiotic administration, and organ dysfunction was assessed by a SOFA score of 2 or more within the 24 h preceding ICU admission, with missing pre-ICU SOFA components set to zero. The source tables and fields used for cohort construction are listed in Supplementary Table S9. Details regarding the Xiaogan electronic health record system and data extraction are available from the corresponding author on reasonable request subject to institutional approval. Among 94,458 ICU admissions of adults from MIMIC-IV (v3.1), 34,216 met the Sepsis-3 criteria for sepsis and 4,241 met the pre-specified SALI biochemical criteria. Of these 4,241 patients, a total of 2,691 were excluded before the final analytical cohort: 1,587 for ICU stay <24 h, 847 for >30% missingness across the pre-specified predictor variables or missing outcome data, and 257 for pre-existing chronic liver disease (cirrhosis, chronic hepatitis, or liver transplant). The remaining 1,550 patients were allocated to a training set (*n* = 1,119) and a held-out internal validation set (*n* = 431; 72% and 28%, respectively). The external validation cohort was established from Xiaogan Central Hospital (January 2018 to October 2025): Of 1,326 screened ICU admissions, 229 met the same pre-specified eligibility criteria after excluding 463 for ICU stay <24 h, 492 for >30% missingness across the pre-specified predictor variables or missing outcome data, and 142 for pre-existing chronic liver disease. Exclusion criteria were age <18 years, ICU stay <24 h, Child–Pugh C chronic liver disease, hepatocellular carcinoma, biliary obstruction, >30% missingness across the pre-specified predictor variables or missing outcome data, and multiple ICU admissions with only the first retained. The 28-day in-hospital mortality rate in the external cohort was 45.9%. The study was approved by the Ethics Committee of Xiaogan Central Hospital (approval No. XGSZXYYLL-EC-2025062501), and informed consent was waived.

### 2.2 Definition of SALI

SALI was defined as total bilirubin >2.0 mg/dL (34.2 µmol/L) and INR >1.5 (23), with both criteria documented within the first 24 h after ICU admission. The first available laboratory measurement within this 24-hour window was used for the SALI eligibility check; no persistence requirement across repeated measurements was imposed. The first-24-h rule was implemented in the extraction code used for the original submission; the 24–72-h window described in the earlier version of the manuscript was a reporting error in the text rather than a change in the eligibility rule. The analytical cohort was therefore not reconstructed and no analysis was re-run: The SALI count (*n* = 4,241), the analytical cohort (*n* = 1,550), and all reported model results are unchanged from the original submission.

### 2.3 Candidate features and outcomes

Thirty-one first-24-h candidate predictors were used in the downstream model analysis script: demographic factors (age, sex); vital signs (peak body temperature, heart rate, respiratory rate, mean arterial pressure [MAP], diastolic blood pressure [DBP], oxygen saturation [SpO_2_]); complete blood count (white blood cells, red blood cells [RBCs], haemoglobin, haematocrit [Hct], platelets); liver function tests (ALT, AST, ALP, albumin, total bilirubin, INR); kidney function tests (blood urea nitrogen, creatinine); electrolytes (sodium, potassium, calcium, chloride, bicarbonate); metabolic parameters (glucose, lactate); and therapeutic variables [mechanical ventilation, vasopressor use, continuous renal replacement therapy (CRRT)]. In the extraction script, laboratory and vital sign variables were summarised according to feature-specific aggregation rules within the first 24 h; SBP, DBP, and MAP were summarised by their 24-h means, whereas other continuous variables followed the documented maximum or minimum rules. Unit conversions and explicit temperature/BMI range exclusions were applied during cohort export. The artefact-as-missing sensitivity analysis was kept separate from the primary pre-processing pipeline. Binary treatment variables were encoded as 0/1 indicators in the model analysis dataset. The primary outcomes were all-cause in-hospital mortality within 7, 14, and 28 days after ICU admission, with deaths ascertained only during index hospitalisation. Index hospitalisation was the hospital admission during which the patient first met the SALI criteria; where a patient had more than one eligible ICU stay, the stay with the earliest ICU admission time was retained and all later ICU stays and later admissions were discarded, so that each patient contributed one record. An event at a given horizon therefore required the in-hospital death timestamp to fall inside index hospitalisation and within the horizon of index ICU admission, and a death recorded only in the out-of-hospital vital status linkage could not create an event. In MIMIC-IV, in-hospital death was identified from the hospital expiration indicator (hospital_expire_flag, mimiciv_hosp.admissions) and timed from the in-hospital death timestamp (deathtime, mimiciv_hosp.admissions); a horizon-specific event was recorded when the death timestamp fell within 7, 14, or 28 days of ICU admission. The linked out-of-hospital vital status date (dod, mimiciv_hosp.patients, derived from the Social Security Death Master File) was not used to define the primary endpoint, because it may have captured deaths occurring after discharge from the index hospitalisation; it was used only in the sensitivity analysis described in Section 2.10. Patients discharged alive before the corresponding horizon were classified as survivors, so no post-discharge death contributed to any endpoint. In the Xiaogan external cohort, the same estimand was ascertained from discharge records only, without linkage to external vital status registries. Field-level provenance for both cohorts is provided in Supplementary Table S9. Complete variable definitions, units, sampling windows, aggregation rules, and missingness rates are provided in Supplementary Table S1.

### 2.4 Data pre-processing

Participant-level missing data exclusions were applied during cohort construction using the pre-specified rule of >30% missingness across the pre-specified predictor variables or missing outcome data. This rule was distinct from feature-level missingness assessed before modelling. Median imputation and Z-score standardisation were estimated in the MIMIC training cohort and then applied unchanged to the internal and external validation cohorts. Thus, the residual missingness shown in Supplementary Table S1 refers to the retained cohort before imputation. The held-out internal validation and external validation sets were not used to estimate the missing data parameters.

### 2.5 Modified SOFA and MELD-Na scores

Because PaO_2_/FiO_2_ was unavailable, a binary modified SOFA score excluding the respiratory component was used. The retained components were coagulation (+1 if platelets <100 × 10⁹/L), liver (+1 if total bilirubin >34.2 µmol/L), cardiovascular (+1 if MAP <70 mmHg or any vasopressor use), central nervous system (+1 if total Glasgow Coma Scale [GCS] < 13), and renal (+1 if creatinine >106 µmol/L). Each component was coded 0/1, giving a total modified score range of 0–5. Vasopressor use was defined as any use within the first 24 h; no dose-specific threshold was applied. In GCS sensitivity analysis, MIMIC total GCS was reconstructed from eye, verbal, and motor components observed within the first 24 h, with the lowest observed total used for the revised score. The external cohort already contained total GCS, so its revised modified SOFA value was unchanged. MELD-Na was calculated using the standard formula, with sodium constrained to 125–140 mmol/L.

### 2.6 Candidate algorithms and feature selection

The candidate feature selection methods were SelectKBest, mutual information, chi-square, RFE-RF, RFE-LR, LASSO, tree importance, gradient-boosting importance, variance threshold, and no selection. The candidate classifiers were logistic regression [LR], random forest, XGBoost, LightGBM, support vector machine, k-nearest neighbours, naive Bayes, decision tree, extra trees, and multilayer perceptron. Each method was combined with each classifier, giving 100 candidate pipelines. Each horizon was modelled independently.

### 2.7 Cross-validation and hyperparameter optimisation

Stratified 10-fold cross-validation was used to evaluate the 100 candidate pipelines within the 1,119-patient MIMIC training cohort, followed by assessment on the held-out internal validation set (24, 25). The final model settings are listed in Supplementary Table S4. The candidate pipelines comprised ten feature selection methods×ten classifiers, with each horizon modelled independently. The final reported models were LightGBM for 7-day mortality and logistic regression for 14- and 28-day mortality; all three final models used the 31 candidate predictors. The final models were fitted using the MIMIC training cohort and evaluated on the separate internal validation set (*n* = 431), followed by application without updating to the external cohort (*n* = 229).

### 2.8 External validation, re-calibration, and statistical comparisons

For each horizon, the corresponding final model was applied to the external cohort without updating. Logistic recalibration of the 14-day model was then performed on the same external cohort as an exploratory model-updating analysis; its post-recalibration performance was not treated as independent external validation. Class-imbalance handling was implemented at the classifier level: The final 7-day LightGBM and the final 14- and 28-day logistic regression models used “class_weight = balanced”; no over-sampling or under-sampling was performed. Other candidate classifiers in the 100-pipeline analysis used their predefined class-balancing parameters where specified in the code. AUROC confidence intervals were estimated by 1,000 bootstrap replicates, and paired AUROCs were compared using a correlated AUROC comparison procedure (26). Among the AUROC comparisons, the primary confirmatory comparison was the 28-day model versus modified SOFA; all other time horizon comparisons (7-day and 14-day), comparisons versus MELD-Na, and comparisons involving the parsimonious logistic regression model were considered exploratory. Brier score, calibration slope and intercept, AUPRC, and classification metrics at a pre-specified threshold of 0.5 were also evaluated.

### 2.9 SHAP explainability analysis

The final model for each horizon was interpreted with a model-matched SHAP method (27, 28). TreeExplainer was used for the tree-based 7-day LightGBM model, and KernelExplainer for the 14- and 28-day logistic regression models. Global importance was summarised by mean absolute SHAP value. SHAP analyses were interpreted within each model and prediction horizon. Differences in SHAP magnitude or ranking across horizons were considered descriptive and hypothesis-generating rather than directly comparable across model classes. SHAP values represent model attribution rather than causal effects. All reported SHAP attributions were computed from the final fitted models that generated the reported predictions. The pre-processing parameters and the coefficient vectors and intercepts required to recompute those predictions are reported in Sections A and B of Supplementary Table S8, and the analysis code, the cohort extraction script, and the pre-processing parameters are publicly available (Data Availability Statement). The coefficients apply to standardised predictors, so their magnitudes are not directly comparable across features, and per-feature SHAP rankings should be read from Table 1, Figure 1, and Supplementary Table S3 rather than inferred from coefficient magnitude.

**Table 1:** Top-five SHAP features by prediction horizon.

Rank	7-day	14-day	28-day
1	SpO_2_ (0.455)	Haematocrit (0.065)	Haematocrit (0.060)
2	INR (0.344)	Vasopressors (0.064)	Vasopressors (0.051)
3	Lactate (0.313)	MAP (0.046)	MAP (0.040)
4	ALT (0.289)	Sodium (0.042)	RBC (0.040)
5	CRRT (0.273)	Heart rate (0.027)	Sodium (0.033)

Values are mean |SHAP|. Importance magnitudes are model-dependent and should not be interpreted as directly comparable across different model classes. ALT, alanine aminotransferase; CRRT, continuous renal replacement therapy; INR, international normalised ratio; MAP, mean arterial pressure; RBC, red blood cell; SpO_2_, peripheral oxygen saturation.

**Figure 1:**
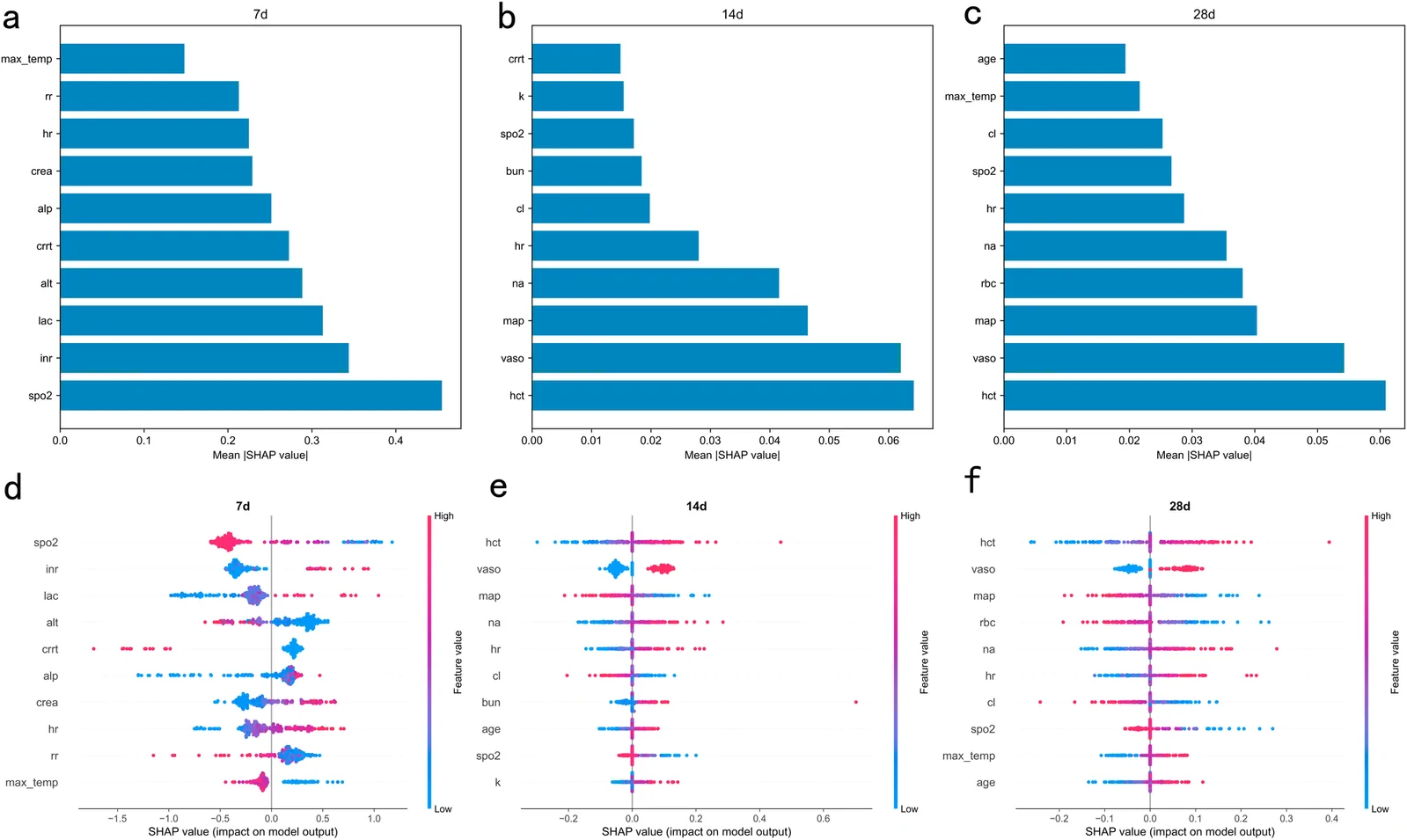
SHAP interpretation of the final models across the three prediction horizons. **(a-c)** Bar plots of mean absolute SHAP values for the 7-, 14-, and 28-day models, respectively. **(d-f)** SHAP summary (beeswarm) plots for the 7-, 14-, and 28-day models, respectively. SpO_2_, INR, and lactate were among the leading contributors for the 7-day model, whereas Hct, vasopressor use, and MAP ranked highly for the 14- and 28-day models.

### 2.10 Robustness and sensitivity analyses

Pre-specified sensitivity analyses were undertaken without changing the primary cohort or primary outcome labels. We (i) reconstructed the modified SOFA CNS component using total GCS; (ii) repeated the score comparison using corrected GCS; (iii) repeated outcome analyses using alternative mortality date labels derived from the external survival file follow-up, which can also capture deaths occurring after discharge, and excluding the 16 external records for which these alternative labels disagreed with the primary index hospitalisation in-hospital label; and (iv) treated clearly implausible monitor values as missing in an evidence-based artefact analysis. These analyses were considered supportive sensitivity analyses rather than replacements for the primary analysis.

## 3 Results

### 3.1 Baseline characteristics

The study design and patient screening are summarised in Figures 2,3. Baseline characteristics of the MIMIC training set and the external validation cohort are shown in Table 2. The two cohorts differed substantially across several variables, including oxygenation, transaminases, lactate, blood counts and use of mechanical ventilation, vasopressors, and CRRT. Continuous variables with a marked right skew were summarised using medians and interquartile ranges, as shown in Table 2. These case-mix differences were considered when interpreting external validation performance.

**Figure 2:**
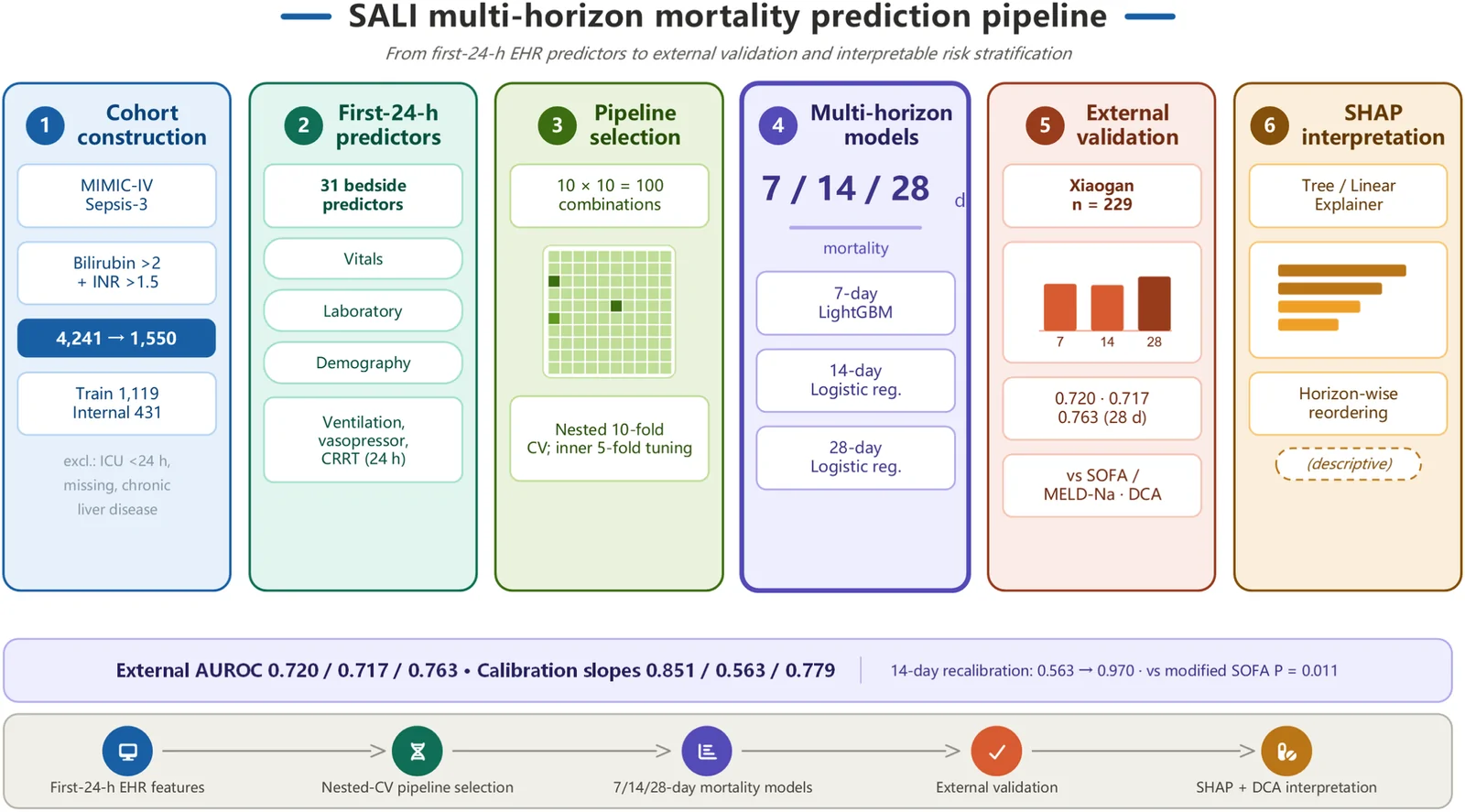
Study design and modelling workflow. The study included an MIMIC-IV (v3.1) development cohort (*n* = 1,550) and an independent Xiaogan Central Hospital external validation cohort (*n* = 229). Thirty-one clinical predictors were assessed within the first 24 h. One hundred candidate pipelines (10 feature selection methods×10 classifiers) were evaluated using stratified 10-fold cross-validation within the training cohort, followed by assessment on a separate held-out internal validation set and the independent external cohort. External comparisons included modified SOFA and MELD-Na, together with calibration, decision curve analysis, and model-matched SHAP interpretation.

**Figure 3:**
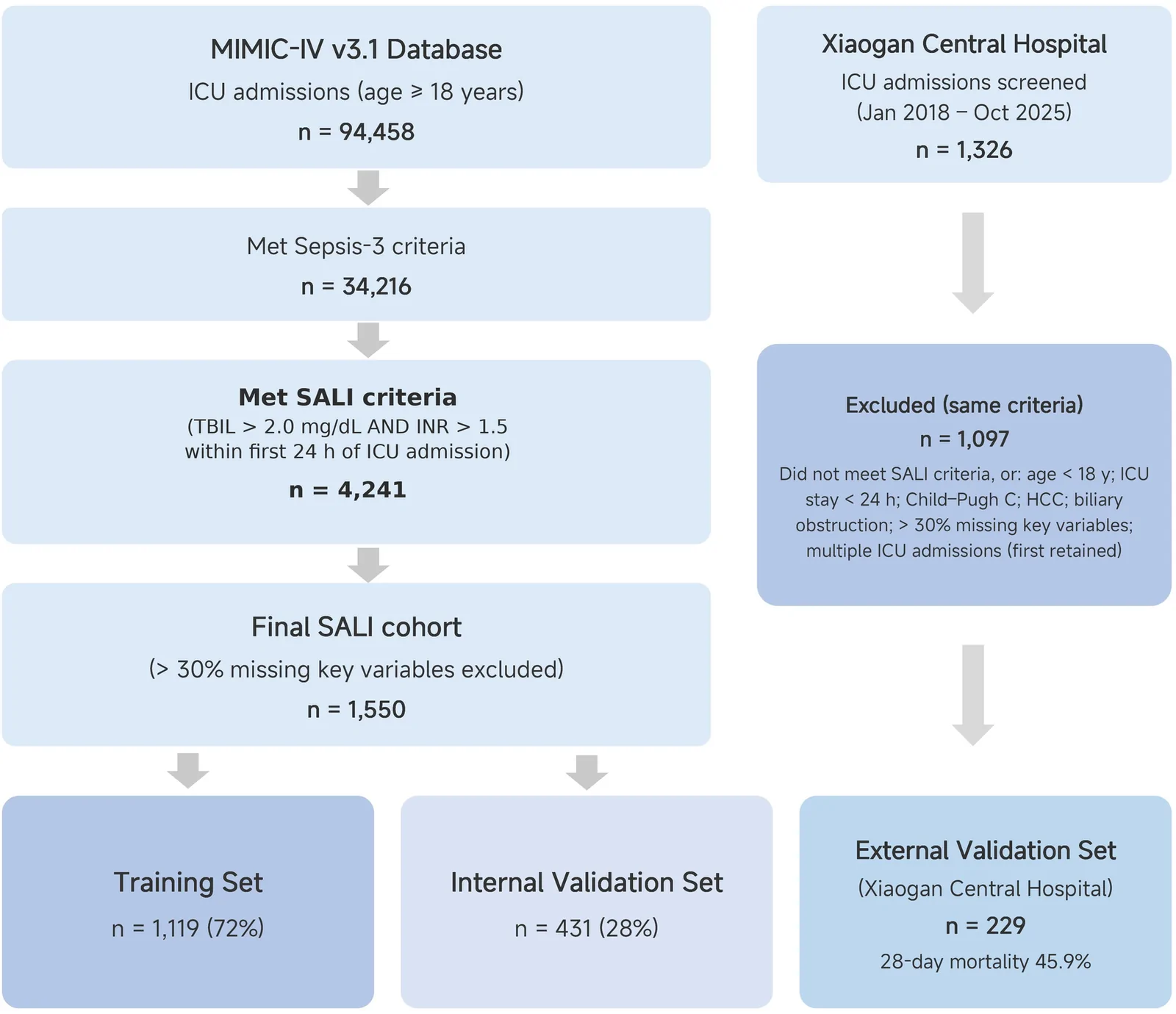
Patient screening flowchart. Screening of 94,458 MIMIC-IV (v3.1) adult ICU admissions yielded 34,216 sepsis cases and 4,241 cases meeting the pre-specified SALI criteria (total bilirubin >2.0 mg/dL and INR >1.5 within the first 24 h of ICU admission). After applying exclusion criteria (1,587 for ICU stay <24 h, 847 for >30% missingness across the pre-specified predictor variables or missing outcome data, and 257 for pre-existing chronic liver disease), 1,550 patients remained and were allocated to training (*n* = 1,119; 72%) and held-out internal validation (*n* = 431; 28%). The independent Xiaogan cohort comprised 1,326 screened ICU admissions, with 229 retained after the same pre-specified eligibility criteria (463 excluded for ICU stay <24 h, 492 for >30% missingness across the pre-specified predictor variables or missing outcome data, and 142 for pre-existing chronic liver disease); 28-day in-hospital mortality was 45.9%.

**Table 2:** Baseline characteristics of the MIMIC-IV training set and the external validation cohort.

Characteristic	MIMIC-IV training (*n* = 1,119)	External validation (*n* = 229)	*P*-value
Age (years)	55.5 ± 14.3	64.4 ± 14.7	<0.001
Male (%)	61.2	56.3	0.193
Peak temperature ( °C)	37.8 ± 0.9	38.3 ± 1.5	<0.001
Heart rate (bpm)	122.3 ± 23.6	116.9 ± 23.8	<0.001
Respiratory rate (min⁻^1^)	33 (28–40)	25 (22–31)	<0.001
MAP (mmHg)	74.9 ± 10.8	67.9 ± 12.4	<0.001
Diastolic BP (mmHg)	60.8 ± 9.8	55.2 ± 11.1	<0.001
SpO_2_ (%)	82.6 ± 17.5	89.3 ± 11.3	<0.001
WBC (×10⁹/L)	16.3 ± 11.3	14.8 ± 9.9	0.130
RBC (×10^12^/L)	3.0 ± 0.7	3.6 ± 0.9	<0.001
Haemoglobin (g/L)	86.6 ± 21.5	111.0 ± 27.3	<0.001
Haematocrit (%)	26.1 ± 6.5	33.8 ± 8.2	<0.001
Platelets (×10⁹/L)	104.8 ± 84.2	128.7 ± 95.6	<0.001
ALT (U/L)	62 (27–319)	37 (17–104)	<0.001
AST (U/L)	136 (58–609)	50 (24–143)	<0.001
ALP (U/L)	174.3 ± 184.4	161.6 ± 148.6	0.448
Albumin (g/L)	28.4 ± 6.9	31.6 ± 6.2	<0.001
Total bilirubin (µmol/L)	97.5 (58.2–194.0)	37.2 (34.3–51.9)	<0.001
INR	2.8 ± 1.7	1.9 ± 1.4	<0.001
BUN (mmol/L)	12.1 (7.14–19.6)	10.0 (6.69–16.9)	0.020
Creatinine (µmol/L)	150 (88.4–265)	118 (85–208)	0.003
Sodium (mmol/L)	134.9 ± 6.4	138.2 ± 6.1	<0.001
Potassium (mmol/L)	3.9 ± 0.7	4.1 ± 0.9	0.764
Calcium (mmol/L)	2.0 ± 0.3	2.1 ± 0.3	<0.001
Chloride (mmol/L)	99.5 ± 7.9	101.9 ± 6.8	<0.001
Bicarbonate (mmol/L)	18.5 ± 5.5	20.8 ± 4.2	<0.001
Glucose (mmol/L)	9.8 ± 5.0	8.4 ± 4.7	<0.001
Lactate (mmol/L)	3.7 (2.2–6.5)	2.1 (1.9–2.25)	<0.001
Mechanical ventilation (%)	71.2	22.7	<0.001
Vasopressor use (%)	60.1	39.7	<0.001
CRRT (%)	14.5	5.7	<0.001

Continuous variables are presented as mean ± SD or median (IQR), as appropriate; categorical variables are presented as percentages. Standardised differences were evaluated as a sensitivity description of development-versus-validation case mix differences. ALT, alanine aminotransferase; ALP, alkaline phosphatase; AST, aspartate aminotransferase; BP, blood pressure; BUN, blood urea nitrogen; CRRT, continuous renal replacement therapy; INR, international normalised ratio; MAP, mean arterial pressure; RBC, red blood cell; SpO_2_, peripheral oxygen saturation; WBC, white blood cell.

### 3.2 Internal validation performance

The internal validation performance of the final models is summarised in Table 2. Supplementary Table S8 provides the pre-processing parameters, complete coefficient vectors and intercepts for the 14- and 28-day logistic-regression models, and the full parameterisation of the 7-day LightGBM model. These entries define the reported final model specifications and are provided to facilitate independent reproduction. These entries therefore define the final model specifications rather than later reproducibility refits. No reported manuscript estimate was changed in this revision.

### 3.3 External validation discrimination and calibration

External validation performance is summarised in Table 4. AUROCs were 0.720 (95% CI 0.643-0.795) at 7 days, 0.717 (0.650-0.784) at 14 days, and 0.763 (0.702-0.825) at 28 days. The internal and external validation performance of the 28-day model is shown in Figure 4, while Figure 5 presents AUROC across the 100 candidate pipelines for the 28-day horizon. The 28-day model showed better discrimination than modified SOFA (0.673, 0.605–0.736; paired AUROC comparison *P* = 0.011) and MELD-Na (0.636, 0.564–0.703; *P* = 0.002). At 7 and 14 days, the differences against SOFA were not statistically significant (*P* = 0.660 and *P* = 0.088, respectively). The difference against MELD-Na was significant at 14 days (*P* = 0.017) but not at 7 days (*P* = 0.132). The corrected-GCS fair-baseline sensitivity model is presented separately in Section 3.5.

**Table 3:** Internal validation performance of the final models.

Horizon	Final feature selection	Final algorithm	10-fold CV AUROC (SD)	Internal validation AUROC
7-day	All features	LightGBM	0.829 (0.027)	0.840
14-day	All features	Logistic regression	0.813 (0.029)	0.841
28-day	All features	Logistic regression	0.818 (0.025)	0.850

The 100 candidate pipelines were evaluated using stratified 10-fold cross-validation within the training cohort; the final model specifications are reported in Supplementary Table S4.

**Table 4:** External validation performance of the final models.

Horizon	ML AUROC (95% CI)	SOFA AUROC (95% CI)	MELD-Na AUROC (95% CI)	*P* (ML vs. SOFA)	*P* (ML vs. MELD-Na)
7-day	0.720 (0.643–0.795)	0.700 (0.625–0.770)	0.638 (0.564–0.709)	0.660	0.132
14-day	0.717 (0.650–0.784)	0.654 (0.579–0.722)	0.612 (0.539–0.683)	0.088	0.017
28-day	0.763 (0.702–0.825)	0.673 (0.605–0.736)	0.636 (0.564–0.703)	0.011	0.002

Pairwise AUROC comparisons were performed using the pre-specified correlated AUROC comparison procedure described in Section 2.8.

**Figure 4:**
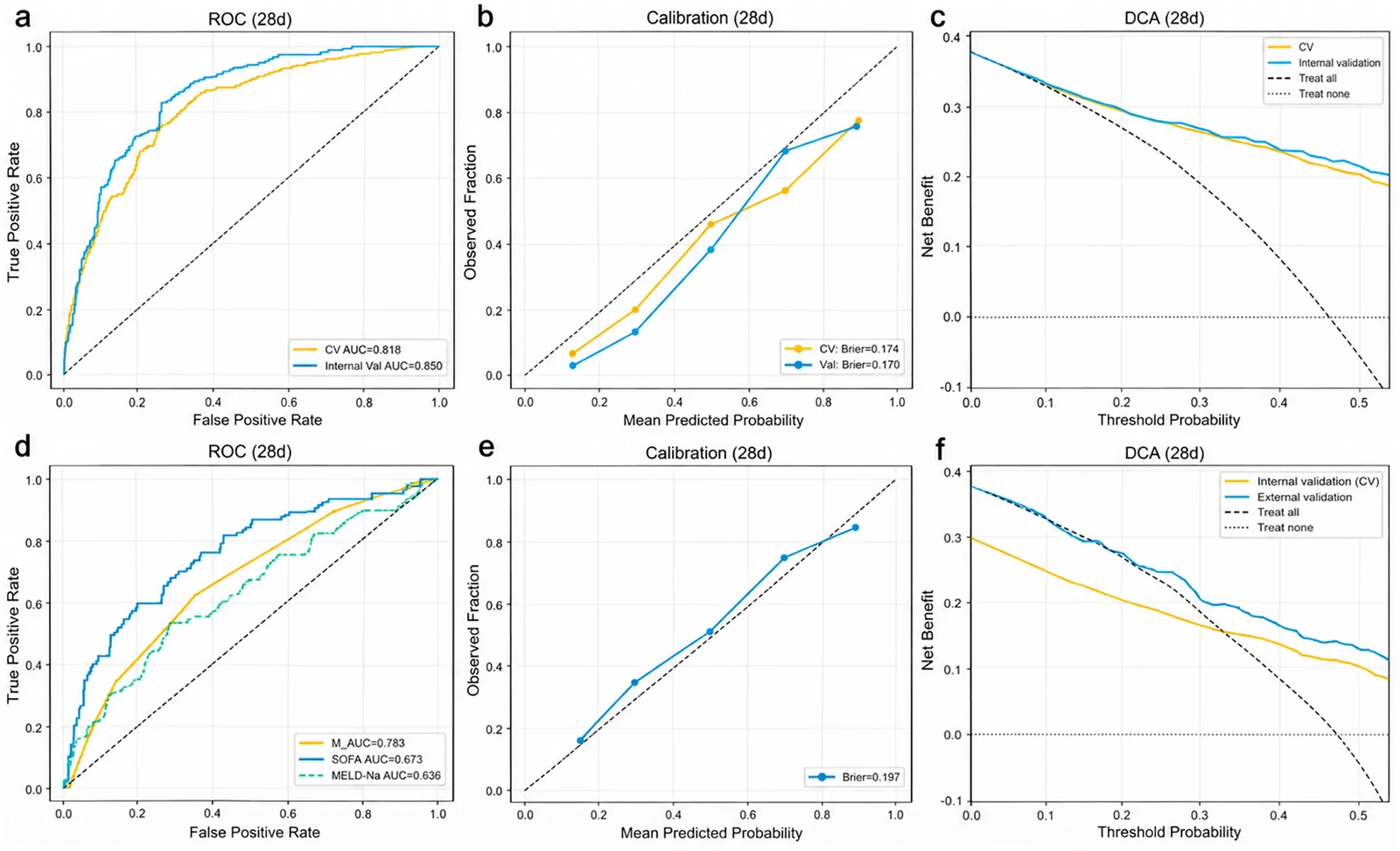
Internal and external validation performance of the 28-day model. **(a)** Receiver operating characteristic (ROC) curves for 10-fold cross-validation and held-out internal validation in the MIMIC-IV development cohort. **(b)** Calibration curves for cross-validation and internal validation. **(c)** Decision curve analysis for cross-validation and internal validation. **(d)** External ROC curves comparing the machine-learning model with modified SOFA and MELD-Na. **(e)** Calibration of the final model in the external cohort. **(f)** Decision curve analysis comparing internal and external validation. The cross-validation AUROC was 0.818, the held-out internal validation AUROC was 0.850, and the external AUROC was 0.763 for the machine-learning model, compared with 0.673 for modified SOFA and 0.636 for MELD-Na. The Brier score in the external cohort was 0.197. Corresponding plots for the 7- and 14-day models are provided in Supplementary Figures S1–S4.

**Figure 5:**
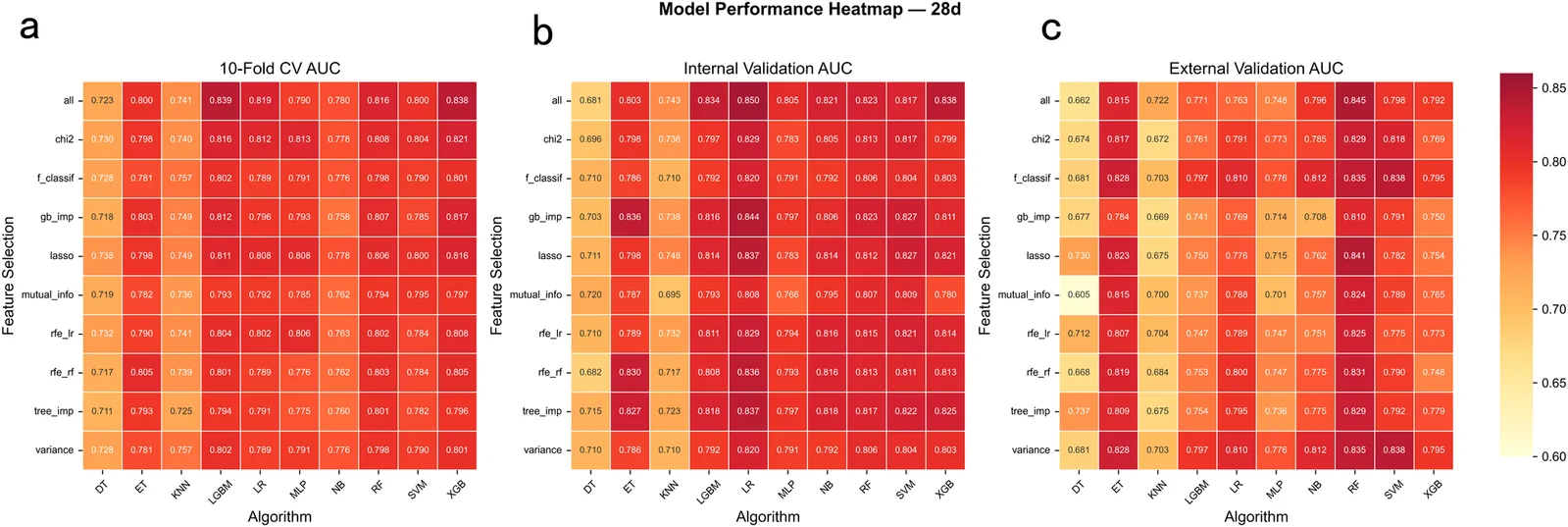
Performance heatmap of the 100 candidate pipelines for the 28-day horizon. AUROC for every combination of feature selection method (rows: all, chi2, f_classif, gradient-boosting importance, LASSO, mutual information, RFE-LR, RFE-RF, tree importance, variance threshold) and classifier (columns: decision tree, extra trees, k-nearest neighbours, LightGBM, logistic regression, multilayer perceptron, naive Bayes, random forest, support vector machine, XGBoost) in **(a)** 10-fold cross-validation, **(b)** held-out internal validation, and **(c)** external validation. Corresponding heatmaps for the 7- and 14-day horizons are provided in Supplementary Figures S5 and S6.

Calibration slopes in the external cohort were 0.851 (95% CI 0.64–1.06), 0.563 (95% CI 0.32–0.81), and 0.779 (95% CI 0.58–0.98) for the 7-, 14-, and 28-day models, respectively. The corresponding calibration intercepts were 0.33 (95% CI −0.23 to 0.88) at 7 days and 0.08 (95% CI −0.42 to 0.59) at 28 days. For the 14-day model, the calibration intercept was −0.178 before recalibration and approximately −0.01 after recalibration; the slope increased from 0.563 to 0.970. The Brier score remained essentially unchanged (0.208 before vs. 0.209 after), indicating correction of slope without meaningful improvement in overall probabilistic accuracy. Because recalibration was fitted and evaluated on the same external cohort, this process is considered an exploratory model updating step rather than independent validation. Decision curve analysis showed positive net benefit across clinically relevant threshold probabilities; exact threshold-specific values are provided in Supplementary Table S5 rather than relying on an isolated single-threshold gain statement. AUPRC values for the external cohort were 0.635 (7-day), 0.647 (14-day), and 0.734 (28-day); 95% bootstrap confidence intervals were 0.498–0.755, 0.515–0.757, and 0.630–0.817, respectively. Brier scores in external validation were 0.185 (7-day), 0.208 (14-day), and 0.197 (28-day); 95% bootstrap CI were 0.142–0.232, 0.167–0.252, and 0.155–0.242, respectively. Summary classification metrics are provided in Supplementary Table S6.

### 3.4 Model interpretation via SHAP

SHAP-derived feature rankings for the three final models differed across prediction horizons (Figure 1). In the primary analysis, SpO_2_, INR, and lactate ranked highest for the 7-day model, whereas Hct, vasopressor use, and MAP ranked highly for the 14- and 28-day models. SHAP analyses were interpreted within each model and prediction horizon. Differences in SHAP magnitude or ranking across horizons were considered descriptive and hypothesis-generating rather than directly comparable across model classes. SHAP values represent model attribution rather than evidence of changing mechanisms.

### 3.5 Sensitivity analyses

Several sensitivity analyses supported the stability of the primary external findings. Reclassification using the survival-file-derived mortality date sensitivity labels increased external AUROC to 0.747, 0.765, and 0.799 at 7, 14, and 28 days, respectively, and excluding the 16 discordant external records yielded 0.745, 0.759, and 0.804. The 16 discordant records were identified by disagreement between the primary in-hospital outcome label and the alternative survival-file-derived mortality date label used in the sensitivity analysis. Because the external analytic export did not retain identifiers and source timestamps sufficient for independent record-level adjudication, the primary labels were retained for the main analysis, and the exclusion analysis is reported only as a robustness check. These discrepancies arise by construction rather than from error: The survival file labels incorporate post-discharge vital status, whereas the primary label counts only deaths occurring during index hospitalisation. Retaining the primary labels therefore preserves a single estimand across the development and external cohorts; adopting the survival file labels in the external cohort alone would have made the two cohorts target different outcomes. Treating clearly implausible values as missing produced small, generally favourable changes, with the 28-day external AUROC increasing from 0.763 to approximately 0.771. The corrected GCS parsimonious logistic regression comparator achieved external AUROCs of 0.759, 0.749, and 0.762 at 7, 14, and 28 days; for 28-day mortality, it was not statistically different from the full model (0.762 vs. 0.763; DeLong *P* = 0.970).

## 4 Discussion

### 4.1 Summary of key findings

The primary analysis yielded cross-validation AUROCs of 0.829 (SD 0.027), 0.813 (SD 0.029), and 0.818 (SD 0.025) at 7, 14, and 28 days, respectively. AUROCs on the held-out internal validation set were 0.840, 0.841, and 0.850, respectively. The final specifications retained all 31 predictors and used LightGBM for 7-day mortality and logistic regression for 14- and 28-day mortality. Event counts were 257, 348, and 418 deaths at 7, 14, and 28 days in the training set; 90, 134, and 161 in the held-out internal validation set; and 76, 94, and 105 in the external cohort, respectively. In the external cohort, the 28-day model showed better discrimination than modified SOFA and MELD-Na, whereas a properly specified parsimonious SOFA component logistic regression showed similar discrimination to the full model.

### 4.2 Why three prediction horizons

External AUROCs were 0.720 (95% CI 0.643–0.795) at 7 days, 0.717 (0.650–0.784) at 14 days, and 0.763 (0.702–0.825) at 28 days. The 28-day model showed better discrimination than modified SOFA (0.673, 0.605–0.736; paired AUROC comparison *P* = 0.011) and MELD-Na (0.636, 0.564–0.703; *P* = 0.002). At 7 and 14 days, differences against SOFA were not statistically significant; the 14-day comparison against MELD-Na remained significant (*P* = 0.017). Calibration slopes in the external cohort were 0.851, 0.563, and 0.779 for the 7-, 14-, and 28-day models, respectively. For the 14-day model, the calibration slope in the external cohort was 0.563 before recalibration and increased to 0.970 after logistic recalibration; the calibration intercept changed from −0.178 to approximately −0.01. The Brier score remained essentially unchanged (0.208 before vs. 0.209 after), indicating no meaningful improvement in overall probabilistic accuracy. Because recalibration was fitted and evaluated on the same external cohort, this analysis should be interpreted as exploratory model updating rather than independent external validation. Decision curve analysis showed potential net benefit across clinically relevant threshold probabilities; threshold-specific values are reported in Supplementary Table S5. A parsimonious corrected GCS logistic regression achieved an external 28-day AUROC of 0.762, essentially matching the full model (0.763).

### 4.3 External performance and generalisability

Comparisons with modified SOFA and MELD-Na should be interpreted as assessments of incremental prognostic information rather than strictly matched comparisons of model complexity. The machine learning models integrated 31 candidate variables, whereas modified SOFA and MELD-Na rely on fewer variables. Thus, the observed differences in discrimination do not establish an inherent advantage of machine learning over these established scores. Rather, they indicate whether the broader feature set used in this study provides additional prognostic information in the studied cohorts.

The drop in AUROC from internal to external validation was 0.066–0.106 across the three models, illustrating the effect of cross-system case mix differences (29, 30). The 28-day external AUROC of 0.763 demonstrates moderate discrimination and a degree of transportability to one independent Chinese centre, but not broad generalisability. First, the ML models used 31 predictors, whereas SOFA and MELD-Na are lower-dimensional clinical scoring systems; these score comparisons therefore address predictive performance rather than complexity-matched equivalence. Second, the modified SOFA score omitted the respiratory component and used a binary, simplified organ component scheme, so it should be interpreted as a modified benchmark rather than conventional full SOFA. Third, a properly specified parsimonious logistic regression model using the SOFA component variables and corrected GCS achieved an external 28-day AUROC of 0.762, essentially matching the full model. Thus, the strongest supported comparative claim is that the model provides better discrimination than the conventional bedside scores in this cohort, not evidence that a larger model is necessarily better than every lower-dimensional regression benchmark (31, 32).

### 4.4 What the SHAP rankings show

The SHAP rankings describe which baseline features contribute most to the prediction of death within each horizon; they do not establish causal effects. The models used a single baseline measurement, so temporal changes in feature contribution within individual patients cannot be inferred. The leading position of Hct in the 14- and 28-day models may reflect capillary leak, fluid accumulation, and marrow suppression in prolonged critical illness (33), but these interpretations remain inferential because direct measures of fluid balance and capillary leak were unavailable. The prominence of INR among the 7-day attributions is consistent with immunothrombosis, in which coagulation activation acts as an intravascular effector of innate immunity rather than solely as a downstream marker of illness severity (34). Vasopressor use and MAP are treatment-conditional variables rather than pure biomarkers. Accordingly, SHAP values should be interpreted as model attribution rather than mechanistic or treatment effects. Differences in SHAP magnitude or ranking across horizons are descriptive and hypothesis-generating.

### 4.5 Relation to prior work

Wen et al. (17) reported a 28-day SALI mortality model trained on MIMIC-IV, broadly compatible with the internal performance observed here. Recent 2026 studies have further expanded the SALI machine learning literature, including a dedicated 28-day mortality study (35), a routine indicator prognostic model (23), and an interpretable model for critically ill patients with liver cirrhosis (36). A recent systematic review of AI models for ICU sepsis outcomes also emphasised that external validation, calibration, prospective evaluation, and workflow-level clinical utility remain recurrent gaps (37). Against this background, the present study is best distinguished by its multi-horizon design and geographically independent Chinese external cohort rather than by claiming that machine learning itself is unprecedented in SALI prediction. The corrected fair-baseline analysis further suggests that model complexity does not guarantee better transportability than a parsimonious regression model. We did not evaluate penalised regression (LASSO, ridge, or elastic net) using the same complete 31-predictor set as a standalone comparator because the primary pipeline selection framework already included LASSO among the ten candidate feature selection methods; penalised regression as a standalone comparator would thus overlap with the pipeline selection procedure. This is noted as a methodological limitation (Section 4.7).

### 4.6 Clinical implications

Two properties matter for potential clinical use beyond discrimination: feasibility and interpretability (38). Most of the 31 predictors are routinely obtainable within the first 24 h of ICU admission, which may facilitate future evaluation of integration into electronic health record workflows (39). Decision curve analysis suggests potential net benefit across clinically relevant threshold probabilities, but this finding remains retrospective and threshold-dependent. SHAP-derived patterns provide model attribution information that may assist interpretation. Because external validation consisted of one independent centre, and no prospective clinical impact assessment was performed, the models should currently be regarded as research-stage risk stratification tools. Prospective multicentre evaluation, recalibration, and assessment of clinical workflow impact are needed before implementation can be considered (40, 41). At any future implementation stage, poor-quality or unavailable inputs should be flagged and handled according to the pre-specified pre-processing rules, and use should be limited to trained healthcare professionals with appropriate clinical oversight.

### 4.7 Limitations

These findings are based on retrospective observational data and model-derived feature attribution. SHAP values reflect associations used by the predictive models and should not be interpreted as causal effects or direct treatment recommendations. Prospective studies are needed to determine whether interventions guided by these predictors can improve patient outcomes. No pre-specified subgroup fairness or health-inequality analysis was undertaken; demographic distributions were compared descriptively, and future multicentre evaluation should assess model performance across relevant sociodemographic groups.

Several limitations should be noted. First, the external validation cohort was a single-centre Chinese sample (*n* = 229), limiting precision and generalisability. Second, because predictors were restricted to the first 24 h after ICU admission, the analysis does not represent a dynamic prediction model using time-varying covariates (42, 43), and the 7-day endpoint represents a relatively short interval from predictor assessment. Third, patients discharged alive before the corresponding time horizon were classified as survivors because post-discharge mortality was not ascertained; the endpoints therefore represent time-limited in-hospital mortality rather than complete 7-, 14-, or 28-day mortality, and the same convention was applied in the development and external cohorts. Fourth, the modified SOFA comparator omits the respiratory component and uses binary components, and should not be interpreted as conventional full SOFA. Fifth, the corrected-GCS sensitivity analysis showed that a parsimonious SOFA-component logistic regression performed similarly to the 28-day full model externally, indicating that greater model complexity did not translate into better external discrimination. Sixth, SHAP rankings are model-dependent, and differences between horizons are descriptive and hypothesis-generating rather than causal. Seventh, the exclusion of patients with pre-existing chronic liver disease (*n* = 257 in MIMIC-IV; *n* = 142 in Xiaogan) restricts the models to acute SALI. Eighth, no formal *a priori* sample size calculation was performed for the external validation, and the resulting uncertainty should be considered when interpreting comparative estimates (44). Ninth, penalised regression (LASSO, ridge or elastic net) using the complete 31-predictor set was not evaluated as a standalone comparator; LASSO was included only as one of the candidate feature selection methods. Tenth, the external validation cohort data could not be deposited in a public repository because of institutional data governance restrictions and are available from the corresponding author on reasonable request subject to institutional approval, whereas the analysis code, the cohort extraction script, the pre-processing parameters, and frozen model objects fitted with the reported hyperparameters have been deposited in a public repository (https://github.com/lizhaopupupu/sali-mimic4-reproducibility). Independent record-level adjudication of the external outcomes was therefore not possible.

## 5 Conclusion

We developed an interpretable machine learning framework for mortality risk stratification in SALI across 7-, 14-, and 28-day horizons and evaluated the final models in an independent Chinese retrospective cohort. The 28-day model showed moderate external discrimination and demonstrated higher AUROC than modified SOFA and MELD-Na in the studied cohort, while properly specified parsimonious SOFA-component logistic regression showed similar discrimination. The 14-day model showed miscalibration and underwent exploratory recalibration on the same external cohort; this updating analysis was not treated as independent validation. SHAP analyses identified horizon-specific model attribution patterns that warrant prospective evaluation. These findings support further validation but do not establish broad clinical generalisability or readiness for clinical deployment.

## Data Availability

Publicly available datasets were analysed in this study. These data can be found here: MIMIC-IV (v3.1): https://physionet.org/content/mimiciv/ (credentialed access required). The external validation cohort from Xiaogan Central Hospital is not publicly available due to institutional data governance restrictions, but can be requested from the corresponding author (Wei Shen, xgsw123@hotmail.com), subject to institutional approval.
