# Supplementary material for: Development and external validation of interpretable machine learning models for 7-, 14-, and 28-day mortality in sepsis-associated liver injury
Source: Front Med (Lausanne). 2026 Oct 16;13:1942203. doi: 10.3389/fmed.2026.1942203 (PMC13719580; doi:10.3389/fmed.2026.1942203)
Supplement: Supplementary file 1 [file Table1.docx]

## Table S1. Definitions, sampling/aggregation rules and confirmed missingness rates for the 31 predictors.

| Feature | Definition | MIMIC Development Cohort Missing (%) | External Validation Cohort Missing (%) | Sampling/Aggregation |
| --- | --- | --- | --- | --- |
| age | Age in years; admission value | 0.0 | 0.0 | Admission |
| gender | Male/Female (categorical) | 0.0 | 0.0 | Admission |
| max_temp | Maximum body temperature in °C | 0.0 | 0.0 | First 24 h; maximum |
| hr | Heart rate in beats per minute | 0.0 | 0.0 | First 24 h; maximum |
| rr | Respiratory rate per minute | 0.0 | 0.0 | First 24 h; maximum |
| map | Mean arterial pressure in mmHg | 1.3 | 0.0 | First 24 h; minimum |
| dbp | Diastolic blood pressure in mmHg | 1.4 | 0.0 | First 24 h; minimum |
| spo2 | Pulse oxygen saturation in % | 0.0 | 0.0 | First 24 h; minimum |
| wbc | White blood cell count (×10⁹/L) | 0.3 | 0.0 | First 24 h; maximum |
| rbc | Red blood cell count (×10¹²/L) | 0.1 | 0.0 | First 24 h; mean |
| hb | Hemoglobin in g/L | 0.2 | 0.0 | First 24 h; minimum |
| hct | Hematocrit in % | 0.1 | 0.0 | First 24 h; minimum |
| plt | Platelet count (×10⁹/L) | 0.1 | 0.0 | First 24 h; minimum |
| alt | Alanine aminotransferase in U/L | 0.6 | 0.0 | First 24 h; maximum |
| ast | Aspartate aminotransferase in U/L | 0.4 | 0.0 | First 24 h; maximum |
| alp | Alkaline phosphatase in U/L | 0.6 | 0.0 | First 24 h; maximum |
| alb | Albumin in g/L | 29.2 | 0.0 | First 24 h; minimum |
| tbil | Total bilirubin in μmol/L | 0.0 | 0.0 | First 24 h; maximum |
| inr | International normalized ratio | 0.0 | 0.0 | First 24 h; maximum |
| bun | Blood urea nitrogen in mmol/L | 0.0 | 0.4 | First 24 h; maximum |
| crea | Creatinine in μmol/L | 0.0 | 0.0 | First 24 h; maximum |
| na | Sodium in mmol/L | 0.0 | 0.0 | First 24 h; minimum |
| k | Potassium in mmol/L | 0.0 | 0.0 | First 24 h; minimum |
| ca | Calcium in mmol/L | 0.6 | 0.9 | First 24 h; minimum |
| cl | Chloride in mmol/L | 0.0 | 0.0 | First 24 h; minimum |
| hco3 | Bicarbonate in mmol/L | 0.0 | 0.4 | First 24 h; minimum |
| glu | Glucose in mmol/L | 0.1 | 0.0 | First 24 h; maximum |
| lac | Lactate in mmol/L | 13.0 | 0.9 | First 24 h; maximum |
| vent | Mechanical ventilation (binary) | 0.0 | 0.0 | Any use in first 24 h |
| vaso | Vasopressor use (binary) | 0.0 | 0.0 | Any use in first 24 h |
| crrt | Continuous renal replacement therapy (binary) | 0.0 | 0.0 | Any use in first 24 h |

Note: Missingness is reported for the final 31 predictors in the development and external validation cohorts before within-training-fold median imputation. Missingness was low for most predictors, while albumin (29.2%) and lactate (13.0%) showed relatively higher missingness in the MIMIC development cohort. Missing values were handled by median imputation within each cross-validation training fold.
