# Supplementary material for: Development and external validation of interpretable machine learning models for 7-, 14-, and 28-day mortality in sepsis-associated liver injury
Source: Front Med (Lausanne). 2026 Oct 16;13:1942203. doi: 10.3389/fmed.2026.1942203 (PMC13719580; doi:10.3389/fmed.2026.1942203)
Supplement: Supplementary file 2 [file Table2.docx]

**Table S2: TRIPOD+AI Checklist**

Completed using the official 11-January-2024 TRIPOD+AI checklist structure. The study includes model development and internal/external evaluation, so D;E items are applicable as specified by TRIPOD+AI.

| **Section/Topic** | **Item** | **D/E** | **Checklist item** | **Reported on page/section** |
| --- | --- | --- | --- | --- |
| TITLE | 1 | D;E | Identify study as developing/evaluating a multivariable prediction model, target population and outcome | Title; p. 1 |
| ABSTRACT | 2 | D;E | See TRIPOD+AI for Abstracts checklist | Abstract; p. 1; separate abstract checklist |
| INTRODUCTION - Background | 3a | D;E | Healthcare context and rationale, including existing models | Introduction; p. 1 |
| INTRODUCTION - Background | 3b | D;E | Target population, intended purpose, care pathway and intended users | Introduction; pp. 1-2 |
| INTRODUCTION - Background | 3c | D;E | Known health inequalities between sociodemographic groups | Discussion/Limitations; no prespecified fairness or health-inequality analysis; future multicentre assessment |
| INTRODUCTION - Objectives | 4 | D;E | Specify study objectives and development/evaluation status | Introduction; p. 2 |
| METHODS - Data | 5a | D;E | Sources for development and evaluation, rationale and representativeness | Methods 2.1; p. 2 |
| METHODS - Data | 5b | D;E | Participant accrual dates and end of follow-up where applicable | Methods 2.1; MIMIC-IV source data 2008-2022; Xiaogan Jan 2018-Oct 2025 |
| METHODS - Participants | 6a | D;E | Setting and centres | Methods 2.1; p. 2 |
| METHODS - Participants | 6b | D;E | Eligibility criteria | Methods 2.1-2.2; pp. 2-3 |
| METHODS - Participants | 6c | D;E | Treatments received and handling if relevant | Methods 2.3 and 2.5; pp. 3-4 |
| METHODS - Data preparation | 7 | D;E | Preprocessing and quality checking | Methods 2.3-2.4; Tables S1/S8 |
| METHODS - Outcome | 8a | D;E | Outcome, time horizon, assessment, rationale and consistency | Methods 2.3; Supplementary Table S9 (MIMIC-IV fields, index hospitalisation); Results 3.5; pp. 3-4 |
| METHODS - Outcome | 8b | D;E | Subjective outcome assessment qualifications if applicable | Not applicable; objective EHR-derived outcome |
| METHODS - Outcome | 8c | D;E | Blinding of outcome assessment | Not applicable; retrospective EHR data |
| METHODS - Predictors | 9a | D | Choice of initial predictors and pre-selection | Methods 2.3; p. 3 |
| METHODS - Predictors | 9b | D;E | Define predictors, timing and measurement | Methods 2.3; Table S1 |
| METHODS - Predictors | 9c | D;E | Subjective predictor assessment qualifications if applicable | Not applicable; objective measurements |
| METHODS - Sample size | 10 | D;E | How study size was determined and sample-size calculation | Methods 2.1; cohort size determined by prespecified eligibility; no separate a priori calculation |
| METHODS - Missing data | 11 | D;E | Missing-data handling and omissions | Methods 2.4; p. 4; Tables S1/S8 |
| METHODS - Analytical methods | 12a | D | Data use and partitioning | Methods 2.1, 2.7; pp. 2, 4 |
| METHODS - Analytical methods | 12b | D | Functional form, transformation, rescaling, standardisation | Methods 2.3-2.4; pp. 3-4 |
| METHODS - Analytical methods | 12c | D | Model type, rationale, model-building, hyperparameters and internal validation | Methods 2.6-2.7; Tables S4/S8 |
| METHODS - Analytical methods | 12d | D;E | Cluster heterogeneity handling if applicable | Not applicable; no cluster-level modelling/heterogeneity analysis |
| METHODS - Analytical methods | 12e | D;E | Performance measures/plots and rationale | Methods 2.8-2.10; Results 3.3-3.5 |
| METHODS - Analytical methods | 12f | E | Model updating arising from evaluation | Methods 2.8; exploratory 14-day recalibration |
| METHODS - Analytical methods | 12g | E | How evaluation predictions were calculated | Methods 2.8; Table S8 |
| METHODS - Class imbalance | 13 | D;E | Why/how imbalance handled and recalibration | Methods 2.8; class_weight=balanced; no sampling |
| METHODS - Fairness | 14 | D;E | Fairness approaches and rationale | Discussion 4.7; no prespecified fairness-specific modelling |
| METHODS - Model output | 15 | D | Output and classification thresholds | Methods 2.8; predicted mortality probabilities; threshold 0.5 only for supplementary classification metrics |
| METHODS - Training vs evaluation | 16 | D;E | Differences between development and evaluation data | Methods 2.1-2.3; Table 1 |
| METHODS - Ethical approval | 17 | D;E | Ethics committee and consent/waiver | Ethics Statement |
| OPEN SCIENCE - Funding | 18a | D;E | Funding source and funder role | Funding |
| OPEN SCIENCE - Conflicts | 18b | D;E | Conflicts/financial disclosure | Conflict of Interest Statement |
| OPEN SCIENCE - Protocol | 18c | D;E | Protocol access or not prepared | No protocol was prepared |
| OPEN SCIENCE - Registration | 18d | D;E | Registration information or not registered | Not registered |
| OPEN SCIENCE - Data sharing | 18e | D;E | Data availability | Data Availability Statement |
| OPEN SCIENCE - Code sharing | 18f | D;E | Analytical code availability | Data Availability Statement; code not publicly deposited; final specifications documented in S4/S8; frozen model objects and code available on reasonable request |
| PATIENT & PUBLIC INVOLVEMENT | 19 | D;E | Patient/public involvement or none | No patient/public involvement |
| RESULTS - Participants | 20a | D;E | Participant flow, outcome events and follow-up | Results 3.1; Figures 1-2 |
| RESULTS - Participants | 20b | D;E | Characteristics by source/setting, dates, predictors, treatments, sample size, events, follow-up, missingness | Results 3.1; Table 1; Table S1 |
| RESULTS - Participants | 20c | E | Comparison of predictor distribution with development data | Results 3.1; Table 1 |
| RESULTS - Model development | 21 | D;E | Participants and events in each analysis | Results 3.2-3.5; Tables 2-3 and S7 |
| RESULTS - Model specification | 22 | D | Full prediction model details for new predictions/third-party evaluation | Tables S4 and S8; frozen model objects and analysis code available from the corresponding author (Data Availability Statement) |
| RESULTS - Model performance | 23a | D;E | Performance estimates with confidence intervals | Results 3.2-3.5; Tables 2-3; S5-S7 |
| RESULTS - Model performance | 23b | D;E | Cluster heterogeneity results if examined | Not examined |
| RESULTS - Model updating | 24 | E | Results of model updating | Results 3.3; exploratory 14-day recalibration |
| DISCUSSION - Interpretation | 25 | D;E | Overall interpretation in context | Discussion 4.1-4.6 |
| DISCUSSION - Limitations | 26 | D;E | Study limitations and effects on bias/uncertainty/generalizability | Discussion 4.7 |
| DISCUSSION - Usability | 27a | D | Handling poor-quality/unavailable inputs | Discussion 4.6; Methods 2.4 |
| DISCUSSION - Usability | 27b | D | User interaction and expertise required | Discussion 4.6; trained healthcare professionals with clinical oversight |
| DISCUSSION - Usability | 27c | D;E | Next steps for applicability/generalizability | Discussion 4.6 and Conclusion |
