# Supplementary material for: Development and external validation of interpretable machine learning models for 7-, 14-, and 28-day mortality in sepsis-associated liver injury
Source: Front Med (Lausanne). 2026 Oct 16;13:1942203. doi: 10.3389/fmed.2026.1942203 (PMC13719580; doi:10.3389/fmed.2026.1942203)
Supplement: Supplementary file 3 [file Table3.docx]

## Table S3: Top 15 SHAP Feature Importance Rankings

### 7d Model

| Rank | Feature | Mean \|SHAP\| |
| --- | --- | --- |
| 1 | spo2 | 0.4549 |
| 2 | inr | 0.3441 |
| 3 | lac | 0.3131 |
| 4 | alt | 0.2885 |
| 5 | crrt | 0.2726 |
| 6 | alp | 0.2517 |
| 7 | crea | 0.2291 |
| 8 | hr | 0.225 |
| 9 | rr | 0.2129 |
| 10 | max_temp | 0.1482 |
| 11 | na | 0.1405 |
| 12 | age | 0.1392 |
| 13 | hct | 0.1385 |
| 14 | map | 0.1134 |
| 15 | tbil | 0.0884 |

### 14d Model

| Rank | Feature | Mean \|SHAP\| |
| --- | --- | --- |
| 1 | hct | 0.0646 |
| 2 | vaso | 0.0644 |
| 3 | map | 0.0456 |
| 4 | na | 0.0421 |
| 5 | hr | 0.0272 |
| 6 | bun | 0.0174 |
| 7 | cl | 0.0174 |
| 8 | age | 0.0169 |
| 9 | spo2 | 0.0165 |
| 10 | k | 0.015 |
| 11 | crrt | 0.0146 |
| 12 | rbc | 0.0141 |
| 13 | lac | 0.0132 |
| 14 | hb | 0.0125 |
| 15 | inr | 0.0112 |

### 28d Model

| Rank | Feature | Mean \|SHAP\| |
| --- | --- | --- |
| 1 | hct | 0.0598 |
| 2 | vaso | 0.0505 |
| 3 | map | 0.0404 |
| 4 | rbc | 0.0396 |
| 5 | na | 0.0326 |
| 6 | hr | 0.0275 |
| 7 | spo2 | 0.0253 |
| 8 | cl | 0.0253 |
| 9 | max_temp | 0.0246 |
| 10 | age | 0.0212 |
| 11 | bun | 0.0157 |
| 12 | crea | 0.0144 |
| 13 | ca | 0.0125 |
| 14 | crrt | 0.0109 |
| 15 | hco3 | 0.0098 |

Note: SHAP values are interpreted within each model and horizon. Differences in magnitude or rank across horizons are descriptive and hypothesis-generating and are not treated as directly comparable across different model classes. SHAP values represent model attribution rather than causal effects.
