# Supplementary material for: Development and external validation of interpretable machine learning models for 7-, 14-, and 28-day mortality in sepsis-associated liver injury
Source: Front Med (Lausanne). 2026 Oct 16;13:1942203. doi: 10.3389/fmed.2026.1942203 (PMC13719580; doi:10.3389/fmed.2026.1942203)
Supplement: Supplementary file 4 [file Table4.docx]

## Table S4: Final-model hyperparameters and implementation details.

| Timepoint | Algorithm | Parameter | Value |
| --- | --- | --- | --- |
| 7d | LGBMClassifier | boosting_type | gbdt |
| 7d | LGBMClassifier | class_weight | balanced |
| 7d | LGBMClassifier | colsample_bytree | 1.0 |
| 7d | LGBMClassifier | importance_type | split |
| 7d | LGBMClassifier | learning_rate | 0.05 |
| 7d | LGBMClassifier | max_depth | 3 |
| 7d | LGBMClassifier | min_child_samples | 20 |
| 7d | LGBMClassifier | min_child_weight | 0.001 |
| 7d | LGBMClassifier | min_split_gain | 0.0 |
| 7d | LGBMClassifier | n_estimators | 200 |
| 7d | LGBMClassifier | n_jobs | -1 |
| 7d | LGBMClassifier | num_leaves | 31 |
| 7d | LGBMClassifier | objective | binary |
| 7d | LGBMClassifier | random_state | 42 |
| 7d | LGBMClassifier | reg_alpha | 0.0 |
| 7d | LGBMClassifier | reg_lambda | 0.0 |
| 7d | LGBMClassifier | subsample | 1.0 |
| 7d | LGBMClassifier | subsample_for_bin | 200000 |
| 7d | LGBMClassifier | subsample_freq | 0 |
| 7d | LGBMClassifier | verbose | -1 |
| 14d | LogisticRegression | C | 0.1 |
| 14d | LogisticRegression | class_weight | balanced |
| 14d | LogisticRegression | dual | False |
| 14d | LogisticRegression | fit_intercept | True |
| 14d | LogisticRegression | intercept_scaling | 1 |
| 14d | LogisticRegression | l1_ratio | 0.0 |
| 14d | LogisticRegression | max_iter | 1000 |
| 14d | LogisticRegression | n_jobs | None |
| 14d | LogisticRegression | random_state | 42 |
| 14d | LogisticRegression | solver | lbfgs |
| 14d | LogisticRegression | tol | 0.0001 |
| 14d | LogisticRegression | verbose | 0 |
| 14d | LogisticRegression | warm_start | False |
| 28d | LogisticRegression | C | 0.1 |
| 28d | LogisticRegression | class_weight | balanced |
| 28d | LogisticRegression | dual | False |
| 28d | LogisticRegression | fit_intercept | True |
| 28d | LogisticRegression | intercept_scaling | 1 |
| 28d | LogisticRegression | l1_ratio | 0.0 |
| 28d | LogisticRegression | max_iter | 1000 |
| 28d | LogisticRegression | random_state | 42 |
| 28d | LogisticRegression | solver | lbfgs |
| 28d | LogisticRegression | tol | 0.0001 |
| 28d | LogisticRegression | verbose | 0 |
| 28d | LogisticRegression | warm_start | False |

**Note:** Values of 0, 0.0, -1, None, False and True are the exact scikit-learn/LightGBM settings fitted by the selection procedure (e.g., reg_alpha = 0.0 and reg_lambda = 0.0 indicate that no L1/L2 regularisation was selected for the 7-day LightGBM model; n_jobs = -1 denotes use of all available cores). None, False and True are literal Python settings, not missing entries. l1_ratio = 0.0 is inert under the lbfgs solver (L2-only penalty) and is reported for completeness.
