# Supplementary material for: Development and external validation of interpretable machine learning models for 7-, 14-, and 28-day mortality in sepsis-associated liver injury
Source: Front Med (Lausanne). 2026 Oct 16;13:1942203. doi: 10.3389/fmed.2026.1942203 (PMC13719580; doi:10.3389/fmed.2026.1942203)
Supplement: Supplementary file 5 [file Table5.docx]

Table S5: Threshold-specific net benefit for the 28-day final model in external validation (n=229).

| Threshold probability | Net benefit: model | Net benefit: treat all | Net benefit: treat none |
| --- | --- | --- | --- |
| 0.05 | 0.4300 | 0.4300 | 0.0000 |
| 0.10 | 0.3988 | 0.3984 | 0.0000 |
| 0.15 | 0.3583 | 0.3630 | 0.0000 |
| 0.20 | 0.3286 | 0.3231 | 0.0000 |
| 0.25 | 0.2897 | 0.2780 | 0.0000 |
| 0.30 | 0.2764 | 0.2265 | 0.0000 |
| 0.35 | 0.2193 | 0.1669 | 0.0000 |
| 0.40 | 0.1994 | 0.0975 | 0.0000 |
| 0.45 | 0.1727 | 0.0155 | 0.0000 |
| 0.50 | 0.1485 | -0.0830 | 0.0000 |

Net benefit = TP/n − FP/n × threshold/(1 − threshold). Treat-none net benefit is zero by definition. Exact threshold-specific values are reported without a separate single-threshold gain claim.
