# Supplementary material for: Development and external validation of interpretable machine learning models for 7-, 14-, and 28-day mortality in sepsis-associated liver injury
Source: Front Med (Lausanne). 2026 Oct 16;13:1942203. doi: 10.3389/fmed.2026.1942203 (PMC13719580; doi:10.3389/fmed.2026.1942203)
Supplement: Supplementary file 6 [file Table6.docx]

Table S6: AUPRC, Brier score, accuracy, sensitivity and specificity at threshold 0.5 in internal and external validation.

| Timepoint | Dataset | AUPRC | Brier score | Accuracy | Sensitivity | Specificity |
| --- | --- | --- | --- | --- | --- | --- |
| 7d | Internal validation (n = 431) | 0.606 | 0.156 | 0.763 | 0.678 | 0.786 |
| 7d | External validation (n = 229) | 0.635 | 0.185 | 0.721 | 0.276 | 0.941 |
| 14d | Internal validation (n = 431) | 0.678 | 0.183 | 0.745 | 0.791 | 0.724 |
| 14d | External validation (n = 229) | 0.647 | 0.208 | 0.707 | 0.511 | 0.844 |
| 28d | Internal validation (n = 431) | 0.728 | 0.170 | 0.773 | 0.801 | 0.756 |
| 28d | External validation (n = 229) | 0.734 | 0.197 | 0.690 | 0.514 | 0.839 |

AUPRC, area under the precision-recall curve; the threshold of 0.5 was fixed for reporting and was not optimized on the external cohort.

**Note:** The low external sensitivity at 7 days (0.276) with high specificity (0.941) reflects the fixed, non-optimised 0.5 classification threshold applied to a cohort with a lower 7-day event rate; it does not indicate a missing or erroneous value. Discrimination and calibration metrics (AUROC, AUPRC, calibration slope/intercept, Brier score) are threshold-independent and are reported as the primary performance measures. Threshold-dependent metrics are provided for completeness only.
