# Supplementary material for: Development and external validation of interpretable machine learning models for 7-, 14-, and 28-day mortality in sepsis-associated liver injury
Source: Front Med (Lausanne). 2026 Oct 16;13:1942203. doi: 10.3389/fmed.2026.1942203 (PMC13719580; doi:10.3389/fmed.2026.1942203)
Supplement: Supplementary file 7 [file Table7.docx]

Note: All analyses in this table are sensitivity analyses and do not replace the primary model results.

| Analysis | 7-day | 14-day | 28-day |
| --- | --- | --- | --- |
| Events, training / internal / external | 257 / 90 / 76 | 348 / 134 / 94 | 418 / 161 / 105 |
| Fold-wise preprocessing effect | ΔAUROC ≤0.004 | ΔAUROC ≤0.004 | ΔAUROC ≤0.004 |
| Survival-file-derived external outcome labels | 0.747 | 0.765 | 0.799 |
| Excluding 16 discordant external records | 0.745 | 0.759 | 0.804 |
| Artifact-as-missing external AUROC | ~0.723 | ~0.724 | ~0.771 |
| Corrected-GCS parsimonious LR external AUROC | 0.759 | 0.749 | 0.762 |
| Common-LR SHAP cross-horizon rank correlation | 7d baseline | 0.78 vs 7d | 0.84 vs 7d; 0.86 vs 14d |
| 28-day corrected-GCS fair-LR vs full ML | — | — | 0.762 vs 0.763; DeLong P=0.970 |

**Note:** Approximate values (prefixed with ~) derive from the artifact-as-missing sensitivity analysis and are rounded to three decimals. An em dash (—) indicates that the analysis is defined only for the 28-day horizon (the corrected-GCS fair-baseline comparison was pre-specified for 28 days) and is not applicable at 7 and 14 days, rather than a missing result. Textual entries such as '7d baseline' denote reference categories against which cross-horizon rank correlations are computed. ΔAUROC ≤ 0.004 denotes the maximum absolute change in AUROC across folds.
