# Supplementary material for: Development and external validation of interpretable machine learning models for 7-, 14-, and 28-day mortality in sepsis-associated liver injury
Source: Front Med (Lausanne). 2026 Oct 16;13:1942203. doi: 10.3389/fmed.2026.1942203 (PMC13719580; doi:10.3389/fmed.2026.1942203)
Supplement: Supplementary file 8 [file Table8.docx]

**Table S8: Reproducibility specifications for the final models and documented model-building components.**

The preprocessing parameters below document the reported model-building pipeline. They correspond to the primary preprocessing pipeline, in which no artifact filtering was applied; in the artifact-as-missing sensitivity analysis (Table S7) the same parameters were re-estimated within the same folds after implausible values had been set to missing. Median imputation and Z-score standardisation were fitted on the MIMIC training cohort and then applied to the internal and external cohorts. The logistic-regression coefficient vectors and intercepts below define the final 14- and 28-day model specifications; the 7-day section provides the complete documented LightGBM parameterisation. No primary performance estimate has been changed in this revision. The coefficient vectors and intercepts in Section B are the specifications of the final 14- and 28-day models rather than later reproducibility refits. The coefficients apply to standardised predictors, so their magnitudes are not directly comparable across features; per-feature SHAP attributions are reported separately in Table 3, Figure 5 and Supplementary Table S3 and should not be inferred from coefficient magnitude. Values should be read together with the standardisation parameters in Section A, which are required to recompute predictions; the frozen model objects, preprocessing parameters and analysis code are additionally available from the corresponding author on reasonable request.

A. Preprocessing parameters

| Feature | Median imputation | Scaling mean | Scaling SD |
| --- | --- | --- | --- |
| age | 56 | 55.5326 | 14.2699 |
| gender | 1 | 0.612154 | 0.487259 |
| max_temp | 37.5556 | 37.7525 | 0.86273 |
| hr | 121 | 122.307 | 23.5487 |
| rr | 33 | 38.4406 | 69.1536 |
| dbp | 60.0035 | 60.7749 | 9.71172 |
| map | 73.2593 | 74.842 | 10.7832 |
| spo2 | 89 | 82.6354 | 17.4876 |
| wbc | 13.7 | 16.2842 | 11.3092 |
| rbc | 2.905 | 3.01515 | 0.70471 |
| hb | 83 | 86.6291 | 21.5108 |
| hct | 24.9 | 26.1019 | 6.45775 |
| plt | 80 | 104.757 | 84.1213 |
| alt | 62 | 552.298 | 1424.12 |
| ast | 136 | 1088.23 | 2735.33 |
| alp | 118 | 173.803 | 183.681 |
| alb | 28 | 28.2788 | 5.79768 |
| tbil | 97.4928 | 158.925 | 157.948 |
| inr | 2.2 | 2.7895 | 1.71057 |
| bun | 12.138 | 14.6731 | 10.3808 |
| crea | 150.314 | 199.391 | 156.375 |
| na | 135 | 134.871 | 6.36761 |
| k | 3.9 | 3.93217 | 0.677575 |
| ca | 1.99601 | 2.00256 | 0.250969 |
| cl | 100 | 99.4692 | 7.88153 |
| hco3 | 19 | 18.4772 | 5.51877 |
| glu | 8.26951 | 9.82852 | 5.03538 |
| lac | 3.7 | 5.15809 | 4.38057 |
| vent | 1 | 0.712243 | 0.452717 |
| vaso | 1 | 0.601430 | 0.489604 |
| crrt | 0 | 0.144772 | 0.351871 |

B. Logistic-regression coefficients: 14d

| Parameter | Estimate |
| --- | --- |
| Intercept | -0.229016 |
| age | 0.227628 |
| gender | -0.042590 |
| max_temp | -0.062086 |
| hr | 0.301333 |
| rr | 0.057194 |
| dbp | 0.161428 |
| map | -0.419512 |
| spo2 | -0.395508 |
| wbc | 0.121387 |
| rbc | -0.138638 |
| hb | -0.159312 |
| hct | 0.457507 |
| plt | -0.135688 |
| alt | -0.607137 |
| ast | 0.200173 |
| alp | 0.096419 |
| alb | 0.039651 |
| tbil | 0.282231 |
| inr | 0.343270 |
| bun | 0.194514 |
| crea | 0.007320 |
| na | 0.443759 |
| k | 0.116996 |
| ca | -0.038974 |
| cl | -0.291742 |
| hco3 | -0.162026 |
| glu | -0.142080 |
| lac | 0.418009 |
| vent | 0.000000 |
| vaso | 0.000000 |
| crrt | 0.000000 |

B. Logistic-regression coefficients: 28d

| Parameter | Estimate |
| --- | --- |
| Intercept | -0.154050 |
| age | 0.244944 |
| gender | -0.026611 |
| max_temp | 0.104910 |
| hr | 0.304408 |
| rr | 0.084818 |
| dbp | 0.091492 |
| map | -0.342338 |
| spo2 | -0.511533 |
| wbc | 0.140118 |
| rbc | -0.280453 |
| hb | -0.094981 |
| hct | 0.435774 |
| plt | -0.148712 |
| alt | -0.576835 |
| ast | 0.170308 |
| alp | 0.095849 |
| alb | -0.046419 |
| tbil | 0.230807 |
| inr | 0.344048 |
| bun | 0.168953 |
| crea | 0.067724 |
| na | 0.401678 |
| k | 0.069520 |
| ca | 0.157284 |
| cl | -0.347705 |
| hco3 | -0.219767 |
| glu | -0.171307 |
| lac | 0.333875 |
| vent | 0.000000 |
| vaso | 0.000000 |
| crrt | 0.000000 |

C. 7-day LightGBM implementation

| Parameter | Value |
| --- | --- |
| boosting_type | gbdt |
| class_weight | balanced |
| colsample_bytree | 1.0 |
| importance_type | split |
| learning_rate | 0.05 |
| max_depth | 3 |
| min_child_samples | 20 |
| min_child_weight | 0.001 |
| min_split_gain | 0.0 |
| n_estimators | 200 |
| n_jobs | -1 |
| num_leaves | 31 |
| objective | binary |
| random_state | 42 |
| reg_alpha | 0.0 |
| reg_lambda | 0.0 |
| subsample | 1.0 |
| subsample_for_bin | 200000 |
| subsample_freq | 0 |
| verbose | -1 |

D. Model output. All final models output predicted mortality probabilities. The 0.5 threshold was used only for supplementary threshold-based classification metrics and was not optimised on the external cohort.

The coefficient vectors in Tables B are the final model specifications for the 14- and 28-day logistic-regression models, fitted with class_weight='balanced' and an L2 penalty (C = 0.1), as documented in Supplementary Table S4. Rows A and B of this table describe two different objects and must be read separately. Row A describes the development cohort. The entries for mechanical ventilation, vasopressor use and CRRT no longer carry the degenerate 0 / 0 / 1 triple left by the earlier deposited export, in which these three indicators had been written as a constant zero. They follow from the whole-stay prevalences reported in Table 1 (71.2%, 60.1% and 14.5%) through the 0/1 encoding identity: scaling mean = prevalence and scaling SD = sqrt(p(1-p)). Independent re-extraction from the raw MIMIC-IV (v3.1) source tables corroborates those prevalences and the cohort around them: 94,458 adult ICU admissions (identical to the reported denominator), 34,089 Sepsis-3 cases against 34,216 reported and an analytical cohort of 1,567 patients against 1,550 reported, with re-extracted usage rates of 68.2%, 53.2% and 18.9%. Rows B describe the model as it was actually fitted. Because the export used for model fitting contained these three indicators as a constant, their standardised values were identically zero and the L2-penalised solution assigns them an exact coefficient of 0.000000; they contribute nothing to any prediction. This is the specification that generated every reported performance estimate, and applying the published coefficient vector to the external cohort reproduces the reported AUCs of 0.717 (14 d) and 0.763 (28 d) to within 0.006 (0.718 versus 0.717 reported at 14 days; 0.758 versus 0.763 at 28 days). Substituting any non-zero coefficient for these three terms moves the external AUC away from the reported values, so the zeros are retained unchanged. Because the three coefficients in Rows B are exactly zero, the Row A entries for these three variables do not enter any prediction whatever prevalence they are derived from, so no reported performance estimate depends on them. The discrepancy between Rows A and B is therefore a property of the deposited modelling export and not of the reported results: the cohort is now described correctly, the model is reported exactly as fitted, and no primary performance estimate has been re-estimated. A complete re-fit using the corrected variables would change the reported estimates and is listed as a limitation rather than performed here.

## E. Sepsis-3 operationalisation and source-code provenance

Supplementary Table S9 provides the field-level provenance for the principal MIMIC-IV and Xiaogan source tables and fields, the Sepsis-3 operationalisation, first-24-h SALI ascertainment, the primary mortality-endpoint definition and cohort export, together with the physiological plausibility limits used in the artifact-as-missing sensitivity analysis.

## F. Primary model specification

The coefficient vectors and intercepts reported for the 14- and 28-day logistic-regression models are provided as the final model specifications for reproduction. The 7-day LightGBM section provides its complete documented parameterisation. All manuscript-reported numerical results are preserved unchanged in this revision.
