# Supplementary material for: Development and external validation of interpretable machine learning models for 7-, 14-, and 28-day mortality in sepsis-associated liver injury
Source: Front Med (Lausanne). 2026 Oct 16;13:1942203. doi: 10.3389/fmed.2026.1942203 (PMC13719580; doi:10.3389/fmed.2026.1942203)
Supplement: Supplementary file 9 [file Table9.docx]

**Supplementary Table S9. Cohort-ascertainment provenance for the development (MIMIC-IV v3.1) and external validation (Xiaogan Central Hospital) cohorts.**

*This table provides the field-level provenance requested for independent replication: Sepsis-3 operationalisation, first-24-h SALI ascertainment, the primary mortality endpoint, the physiological plausibility limits used in the artifact-as-missing sensitivity analysis, and the alternative survival-file mortality-date labels used in sensitivity analyses only. No analysis was re-run to produce this table.*

**A. Sepsis-3 operationalisation**

| **Element** | **MIMIC-IV development cohort** | **Xiaogan external cohort** |
| --- | --- | --- |
| Suspected infection | Combination of a blood-culture order and intravenous antibiotic administration | Same operational rule applied to the electronic health-record export |
| Organ-dysfunction criterion | SOFA score ≥2 attributable to infection | SOFA score ≥2 attributable to infection |
| SOFA assessment window | The 24 h preceding ICU admission | The 24 h preceding ICU admission |
| Baseline organ dysfunction | Components missing before ICU admission were set to zero | Components missing before ICU admission were set to zero |
| Source tables / systems | mimiciv_hosp.admissions; mimiciv_icu.icustays; mimiciv_hosp.microbiologyevents; mimiciv_hosp.prescriptions; mimiciv_hosp.labevents | Xiaogan electronic health-record export (laboratory, microbiology, pharmacy and vital-sign modules) |

**B. First-24-h SALI ascertainment**

| **Element** | **MIMIC-IV development cohort** | **Xiaogan external cohort** |
| --- | --- | --- |
| Biochemical criteria | Total bilirubin >2.0 mg/dL (34.2 µmol/L) AND INR >1.5 | Identical thresholds |
| Ascertainment window | Both criteria documented within the first 24 h after ICU admission | Both criteria documented within the first 24 h after ICU admission |
| Measurement rule | First available measurement within the window; no persistence requirement across repeated measurements | First available measurement within the window; no persistence requirement |
| Wording history | The first-24-h rule was applied by the extraction code used for the original submission; the "24–72 h" description in the earlier manuscript version was a reporting error in the text, not a change in the eligibility rule. The cohort was not reconstructed and no analysis was re-run (n=4,241 SALI; n=1,550 analysed). | Not applicable — the external cohort was extracted with the same first-24-h rule |

**C. Primary mortality endpoint (identical estimand in both cohorts)**

| **Element** | **MIMIC-IV development cohort** | **Xiaogan external cohort** |
| --- | --- | --- |
| Estimand | All-cause in-hospital mortality within 7, 14 and 28 days of ICU admission, restricted to the index hospitalisation | Identical estimand |
| Death indicator | mimiciv_hosp.admissions.hospital_expire_flag | In-hospital death status recorded in the discharge record |
| Death timing | mimiciv_hosp.admissions.deathtime (in-hospital death timestamp) | Discharge or death date recorded in the discharge record |
| Horizon assignment | Event recorded when deathtime − ICU admission time ≤ 7, 14 or 28 days | Event recorded when the recorded death date − ICU admission time ≤ 7, 14 or 28 days |
| Fields deliberately not used for the primary endpoint | mimiciv_hosp.patients.dod (linked out-of-hospital vital status derived from the Social Security Death Master File), because it may capture deaths occurring after discharge from the index hospitalisation | No external vital-status registry linkage was available or used |
| Censoring rule | Patients discharged alive before the corresponding horizon were classified as survivors; no post-discharge death contributed to any endpoint | Identical rule |

**D. Physiological plausibility limits (artifact-as-missing sensitivity analysis only)**

| **Variable** | **Accepted range** | **Action** |
| --- | --- | --- |
| Heart rate | 20–300 beats/min | Values outside the range set to missing |
| Respiratory rate | 4–60 breaths/min | Values outside the range set to missing |
| Peripheral oxygen saturation (SpO₂) | 50–100 % | Values outside the range set to missing |
| Mean arterial pressure | 20–200 mmHg | Values outside the range set to missing |
| Diastolic blood pressure | 10–150 mmHg | Values outside the range set to missing |
| Body temperature | 30–45 °C | Values outside the range set to missing |

*These limits were applied only in the artifact-as-missing sensitivity analysis reported in Supplementary Table S7. They were not part of the primary preprocessing pipeline, whose parameters are reported in Supplementary Table S8A.*

**E. Alternative survival-file mortality-date labels (sensitivity analysis only)**

| **Element** | **MIMIC-IV development cohort** | **Xiaogan external cohort** |
| --- | --- | --- |
| Source of the alternative labels | Not applicable | External survival-file follow-up containing mortality dates |
| Relation to the primary endpoint | Not applicable | Captures deaths occurring after discharge, i.e. outside the primary index-hospitalisation estimand |
| Discordant records | Not applicable | 16 external records for which the survival-file label disagreed with the primary in-hospital label |
| Handling | Not applicable | Primary labels retained; reclassification and exclusion of the 16 records evaluated only as robustness analyses (Table S7) |
